# Variation in physiological host range in three strains of two species of the entomopathogenic fungus *Beauveria*

**DOI:** 10.1371/journal.pone.0199199

**Published:** 2018-07-05

**Authors:** Clara Rohrlich, Isabelle Merle, Issa Mze Hassani, Manon Verger, Michel Zuin, Samantha Besse, Isabelle Robène, Samuel Nibouche, Laurent Costet

**Affiliations:** 1 CIRAD, UMR PVBMT, Saint-Pierre, La Réunion, France; 2 Arysta LifeScience Group, BETEL Réunion, Saint-Benoit, La Réunion, France; 3 Université de la Réunion, UMR PVBMT, Saint-Pierre, La Réunion, France; 4 INRAPE, Moroni, Union des Comores; 5 Arysta LifeScience Group, NPP, Pau, France; University of Innsbruck, AUSTRIA

## Abstract

Knowledge of the host range of a biocontrol agent (BCA) is fundamental. Host range determines the BCA’s economic potential, as well as the possible risk for non-target organisms. Entomopathogenic fungal strains belonging to the genus *Beauveria* are widely used as BCA, but our knowledge of their physiological host range is only partial. The aim of this study was to improve our understanding of the physiological host range of three *Beauveria* strains belonging to two species, *B*. *hoplocheli* and *B*. *bassiana*. We performed laboratory mortality bioassays to assess their pathogenicity and virulence against nine insect pests, belonging to three orders: Lepidoptera, Coleoptera and Diptera. Mortality rate, mean survival time and mycosis rate were used to estimate virulence. Pathogenicity was assessed as the capacity to cause a disease and induce mortality. Virulence was assessed as the severity of the disease based on mortality rate, mean survival time and mycosis rate. The results of this study revealed significant differences in the physiological host range of the three *Beauveria* strains tested. The three strains were pathogenic to all Diptera and Lepidoptera species tested. In the case of the Coleoptera, only the *B*. *hoplocheli* strain was pathogenic to the white grub *Hoplochelus marginalis* and only the *B*. *bassiana* strains were pathogenic to *Alphitobius diaperinus*. The *B*. *hoplocheli* strain was less virulent on Lepidoptera and Diptera than the two *B*. *bassiana* strains. The latter both exhibited very similar virulence patterns. The fact that *B*. *hoplocheli* and *B*. *bassiana* strains have different host ranges means that they can be used as BCA to target different pests. Impacts on non-target insects across multiple orders cannot be ruled out in the absence of ecological host range studies.

## Introduction

Host specificity or host range of an entomopathogenic fungus can be defined as the number and taxonomic diversity of the hosts it can infect [1]. Knowledge of the host range of a biocontrol agent (BCA) is fundamental because host range determines the BCA’s possible risk for the environment [2] and economic potential [3]. These two aspects are somewhat correlated, given that a BCA with a broad host range may be lethal for a wide range of target pests and also potentially for a broad range of non-target species [1, 3]. The ecological host range refers to the range of species that an entomopathogenic fungus infects in field conditions. The physiological host range is the range that the pathogen is able to infect under optimized conditions, determined by laboratory tests [4]. The ecological host range is usually considered to be narrower than the physiological host range and a better estimator of the risk for the environment [4–6]. However, assessing the ecological host range of a BCA is a complex task and can only be achieved once the BCA has been introduced in the environment [4]. Thus, the question of host range is not usually explored fully, which means there are gaps in our knowledge. Generally, the characterization of a BCA’s host range is drawn from our knowledge of the hosts on which the strain was collected in natural conditions and a few laboratory pathogenicity tests [7]. In invertebrate pathology, pathogenicity is defined as the capacity to cause a disease to a given host and virulence is defined as the severity of the disease [8, 9]. Different approaches can be used and combined to estimate the virulence of a pathogen. Dose-mortality experiments determine the median lethal dose or concentration, which causes the death of 50% of the test insects; while single dose time-mortality experiments are used to determine the mean survival time or the median lethal time at which 50% of the test insects have died; and fungal growth on the host cadaver can be checked to ascertain completion of the fungal biological cycle [10]. Reduction of other fitness parameters can also be measured, such as fecundity or offspring survival [4].

Entomopathogenic fungi used as commercial BCA have diverse host ranges. Most are capable of infecting a wide range of hosts, although a few have a narrow host range [11]. Fungi belonging to the Entomophthorales order (Entomophthoromycota phylum) tend to have a narrow ecological and physiological host range limited to a small number of taxonomically related species [12, 13]. They include many obligate biotrophic insect pathogens, like all the species belonging to the Entomophthoraceae family [14]. They are difficult to mass-produce and are used primarily for classical biological control with a view to the permanent establishment of the exotic BCA [15]. In contrast, fungi used in inundative or inoculative strategies, involving the regular release of the BCA, have to be mass-produced. The fungi used in these strategies belong mainly to the Hypocreales order (Ascomycota phylum). They are hemibiotrophic and generally have a broad host range [11]. However, among Hypocreales, differences in host range are often mentioned at the species or strain level.

In the genus *Beauveria*, *B*. *bassiana* (Balsamo) Vuillemin is recognized as a generalist species with a broad ecological host range of more than 700 arthropod species, which covers most orders of the class Insecta [6, 16]. *B*. *brongniartii* (Saccardo) Petch is often claimed to have a more restricted host range, infecting mostly Coleoptera [17–19]. However, this fungus species has been reported to infect insects from at least seven orders in the field [18, 20]. For several other species, such as *B*. *vermiconia* de Hoog & Rao or *B*. *caledonica* Bisset & Widden, the number of strains available in collections is too small to draw conclusions about their host range [21]. To date, the species *B*. *hoplocheli* Robène-Soustrade & Nibouche (formerly described as *B*. *brongniartii* or *B*. *tenella*) has only been isolated in natural conditions from the white grub *Hoplochelus marginalis* (Fairmaire) (Coleoptera: Scarabaeidae) [22]. It is used as a BCA against this pest in Réunion Island [23]. Despite a few preliminary studies, the physiological host range of *B*. *hoplocheli* has not been investigated extensively. In laboratory bioassays, *B*. *hoplocheli* exhibited little or no virulence to *Melolontha melolontha* L. (Coleoptera: Scarabaeidae) [24] and to the mango blossom gall midge *Procontarinia mangiferae* (Felt) (Diptera: Cecidomyiidae), but was pathogenic to the greater wax moth *Galleria mellonella* L. (Lepidoptera: Pyralidae) [25].

Many studies have compared the virulence of several strains of *Beauveria* spp. on a given insect host, especially strains of *B*. *bassiana* [26–32]. Few works have studied the physiological host range of *Beauveria* spp. strains by comparing their pathogenicity and virulence on several insect species. For example, 43 *B*. *bassiana* strains collected worldwide exhibited a strong variation in virulence against eight lepidopteran species [33]. Twenty-nine genetically diverse *B*. *bassiana* strains were pathogenic to nine insect species from five orders, with significantly different levels of virulence [34].

Few studies demonstrate that *Beauveria* strains or species may differ in their physiological host range, despite the importance of these differences regarding their use as BCA. Therefore, the aim of this study was to characterize the physiological host range of three *Beauveria* strains belonging to two species, *B*. *hoplocheli* and *B*. *bassiana*. We tested their pathogenicity and their virulence against nine insect pests, belonging to three orders: Lepidoptera, Coleoptera and Diptera. Mortality rate, mean survival time and mycosis rate were used as estimators of the virulence in single dose mortality bioassays.

## Materials and methods

### *Beauveria* strains and spore suspensions

Two strains of *B*. *bassiana* (I-2960, I-2961) and one strain of *B*. *hoplocheli* (B507), were obtained from Arysta LifeScience. The strains were stored at -80°C using Microbank cryovials (Pro-Lab Diagnostics, Richmond Hill, Canada). Cultures were grown from the cryovial stored strains to prepare spore suspensions for the tests. All cultures were grown at 25°C on potato dextrose agar (PDA) medium until sporulation was observed (three to four weeks). Spore suspensions were prepared by scraping the surface of sporulated cultures and suspending conidia in a sterile solution of 0.05% TWEEN^®^ 80 (Sigma-Aldrich, St. Louis, MO, USA). Conidia suspensions were adjusted to 10^6^ or 10^8^ conidia mL^-1^ using a Malassez hemocytometer. To determine conidia viability and the number of conidia per milliter, 100 μL of the conidia suspension was plated onto PDA, incubated at 25°C and the colony forming units were counted after five days.

### Insects

The pathogenicity of the three *Beauveria* spp. strains was evaluated on nine insect species belonging to three orders: Diptera, Coleoptera and Lepidoptera (Table 1). We performed pathogenicity tests on five fruit flies found in Réunion: the peach fruit fly, *Bactrocera zonata* (Saunders), the Mediterranean fruit fly, *Ceratitis capitata* (Wiedemann), the Mascarene fruit fly, *C*. *catoiri* (Guérin-Méneville) endemic in Réunion, the Indian Ocean cucumber fly, *Dacus demmerezi* (Bezzi) and the melon fly, *Zeugodacus cucurbitae* (Coquillett). Tests were also carried out on the oriental fruit fly, *Bactrocera dorsalis* (Hendel), an invasive species not recorded in Réunion at the time of the study, but present in most islands in the western region of the Indian Ocean, including Comoros [35]. Fruit fly strains of *B*. *zonata*, *C*. *capitata* and *C*. *catoirii* were reared on artificial diets [36] in our laboratory for 138, 17 and 157 generations, respectively. *D*. *demmerezi* and *Z*. *cucurbitae* were reared on zucchini for 22 and 64 generations, respectively. Sugarcane white grub *H*. *marginalis* larvae were collected in the field with permission of the owner from plots of sugarcane (Saint-Benoît, Réunion; 20°59'41.05" S, 55°41'16.63" E) and thyme (Petite-Ile, Réunion; 21°20'3.57" S, 55°33'22.13" E). *H*. *marginalis* larvae were kept in the laboratory in clean soil, fed with pieces of carrot and quarantined for 20 days to ensure that they were free from entomopathogenic fungal infections. The lesser mealworm, *Alphitobius diaperinus* (Panzer) (Coleoptera: Tenebrionidae) strain was collected from a poultry farm with permission of the owner (Saint-André, Réunion; 20°56'52.0" S, 55°39'41.2" E). *A*. *diaperinus* larvae were maintained on wood shavings and fed with poultry feed pellets until treatments. Larvae of *G*. *mellonella* came from a strain reared in our laboratory on an artificial diet adapted from Meyling [37].

**Table 1:** Insects used in bioassays.

Species	Common name	Order	Family	Stage	No. insects per treatment	Conidia suspension (conidia.mL^-1^)
*Bactrocera dorsalis*	Oriental fruit fly	Diptera	Tephritidae	Adult	90	10^6^
*Bactrocera zonata*	Peach fruit fly	Diptera	Tephritidae	Adult	90	10^6^
*Ceratitis capitata*	Mediterranean fruit fly	Diptera	Tephritidae	Adult	90	10^6^
*Ceratitis catoirii*	Mascarene fruit fly	Diptera	Tephritidae	Adult	90	10^6^
*Dacus demmerezi*	Indian Ocean cucurbit fly	Diptera	Tephritidae	Adult	90	10^6^
*Zeugodacus cucurbitae*	Melon fly	Diptera	Tephritidae	Adult	150	10^6^
*Alphitobius diaperinus*	Lesser mealworm	Coleoptera	Tenebrionidae	Larva	90	10^8^
*Hoplochelus marginalis*	Sugarcane white grub	Coleoptera	Scarabaeidae	Larva (3rd instar)	90	10^8^
*Galleria mellonella*	Greater wax moth	Lepidoptera	Pyralidae	Larva	390	10^6^ and 10^8^

### Bioassays

Bioassays were conducted on two insect species at a time, using *G*. *mellonella* for all bioassays. Each bioassay compared four modalities: the three *Beauveria* strains B507, I-2960 and I-2961, and an untreated control. Each modality was carried out on 30 insects. For fruit flies, 12 to 14-day-old adults were used, with 15 males and 15 females. Strain B507 was not tested on *C*. *catoirii*. Each bioassay was repeated three times. The experiments were conducted from May 2015 to August 2016 in the CIRAD laboratory (Réunion). Since *B*. *dorsalis* was not recorded in Réunion at the time of the study, bioassays for this species were conducted in the INRAPE laboratory (Comoros).

Insect contamination was realized by dipping the insects in the conidia suspension for 10 seconds. Adult fruit flies were anaesthetized with CO_2_ prior to treatment and dipped in a suspension at 10^6^ conidia mL^-1^. This concentration is in the range of the LC_50_ (lethal concentration required to kill 50% of the target insects) of several *B*. *bassiana* strains tested on fruit flies [38–40]. Preliminary bioassays showed that using 10^6^ conidia mL^-1^, the mortality was similar to control for *A*. *diaperinus* and *H*. *marginalis* larvae for all strains tested. Therefore, the insects were treated with suspensions at 10^8^ conidia mL^-1^. Untreated control fruit flies were anaesthetized with CO_2_ and then dipped in a 0.05% TWEEN^®^ 80 solution like all other control insects.

After treatment, all insects were kept separately in 125 mL plastic containers (flies and white grubs), and 30 mL plastic containers (*G*. *mellonella* and *A*. *diaperinus* larvae). Adult flies were fed three times a week with a liquid diet containing a 10:1 mix of sucrose and yeast enzymatic hydrolysate (MP Biomedicals, Solon, OH, USA). After dipping, *G*. *mellonella* larvae were fed with a 400 mg piece of beeswax and *A*. *diaperinus* larvae were fed with a few poultry feed pellets. *H*. *marginalis* larvae were kept in sterilized peat and fed once a week with slices of organically grown carrots.

Insect mortality was recorded daily for 30 days for the fruit flies and every other day for the other species. Insects were considered dead if they were unable to produce coordinated movements or showed no response when touched. Cadavers were surface-sterilized in 70% ethanol for five seconds, rinsed in sterile distilled water for five seconds and placed on a sterilized filter paper, moistened with 200 μL sterile distilled water, in a 55 mm vented Petri dish to stimulate external fungal growth. Development of mycosis was checked 10 days after death.

### Data analysis

The aim of the first analysis was to compare the effect of the treatments on two variables used to estimate virulence: mortality rate and mycosis rate. The mycosis rate was calculated as the percentage of cadavers showing external fungal growth out of the total number of tested insects. The experimental design allowed us to compare four treatments on two insect species in each bioassay. The presence of *G*. *mellonella* in each bioassay allowed an estimation of the bioassay effect as a fixed replication effect and the design was thus analysed as a classical factorial design. As two spore suspensions were used at 10^6^ or 10^8^ conidia.mL^-1^, we conducted separate analyses on the bioassays using each of the two doses. The analysis of the mycosis rate for bioassays using a 10^8^ conidia.mL^-1^ could not be performed due to missing data for *G*. *mellonella*. We conducted the analysis with a generalized linear model using a binomial distribution and a logit link with SAS GLIMMIX procedure [41]. The model included insect species, treatments, insect species x treatment interaction, bioassays and bioassays x treatments interaction as fixed factors. To solve some convergence issues, maximum likelihood with Laplace approximation was preferred to the default pseudo-likelihood technique. The insects used in the experiments had a different life span and different developmental stages (adult or larva). Therefore, the mean survival time for control insects varied depending on the insect species. In order to take this factor into account and to ensure that mortality rates were not too high in controls, we analysed the mortality rates when the mortality rate for the control reached 0.2. The insect species x treatments interaction was significant (P < 0.05) for both suspensions at 10^6^ and 10^8^ conidia.mL^-1^. Consequently, we carried out between treatment comparisons with each insect species separately. To do so, we used a generalized linear model with a binomial distribution with the SAS GENMOD procedure, using treatment and bioassays as fixed effects. Pairwise between treatment differences were tested using a likelihood ratio test.

The second analysis focused on survival curves. We used the Kaplan-Meier estimator, a non-parametric statistic, to compare the effects of the three *Beauveria* strains on insect survival within an insect species. Survival curves were modelled using the SAS LIFETEST procedure and a log-rank test was performed to detect significant differences between treatments. Since multiple pairwise comparisons of strains increase the overall type 1 error, Sidak’s correction was applied to adjust the significance thresholds in order to yield an experiment-wise P-value of 0.05. The strains’ virulence was estimated by the mean survival time computed using the SAS LIFETEST procedure.

## Results

### Analysis of mortality rate and Kaplan-Meier survival curves

**Fig 1:**
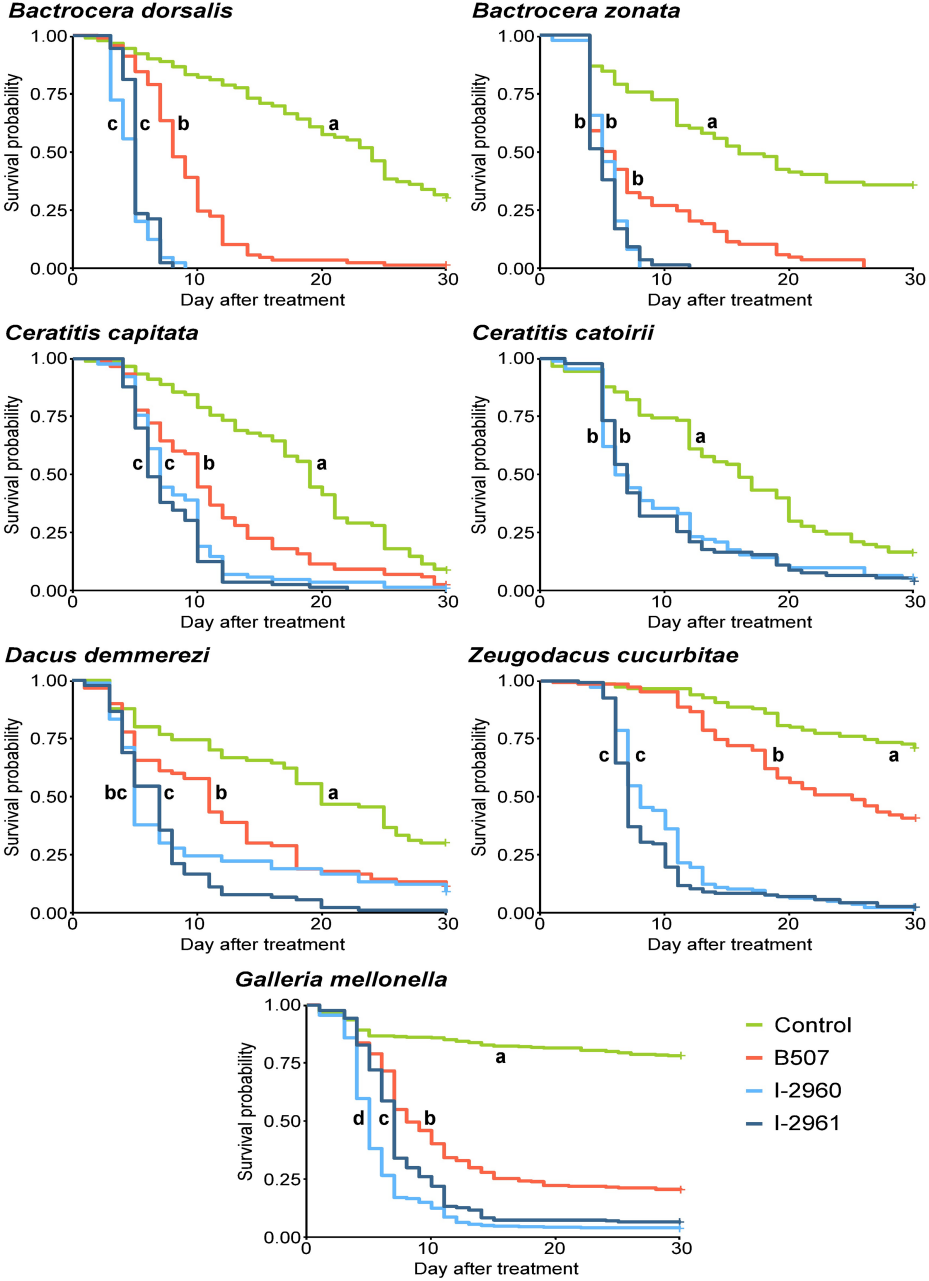
Kaplan-Meier survival curves for six fruit fly species and *Galleria mellonella* treated with *Beauveria hoplocheli* strain B507, *B*. *bassiana* strains I-2960 and I-2961 using 10^6^ conidia.mL^-1^ suspensions. Different letters indicate significant differences between treatments within an insect species (log-rank test, P < 0.05 after Sidak’s correction). Crosses indicate censored data.

**Fig 2:**
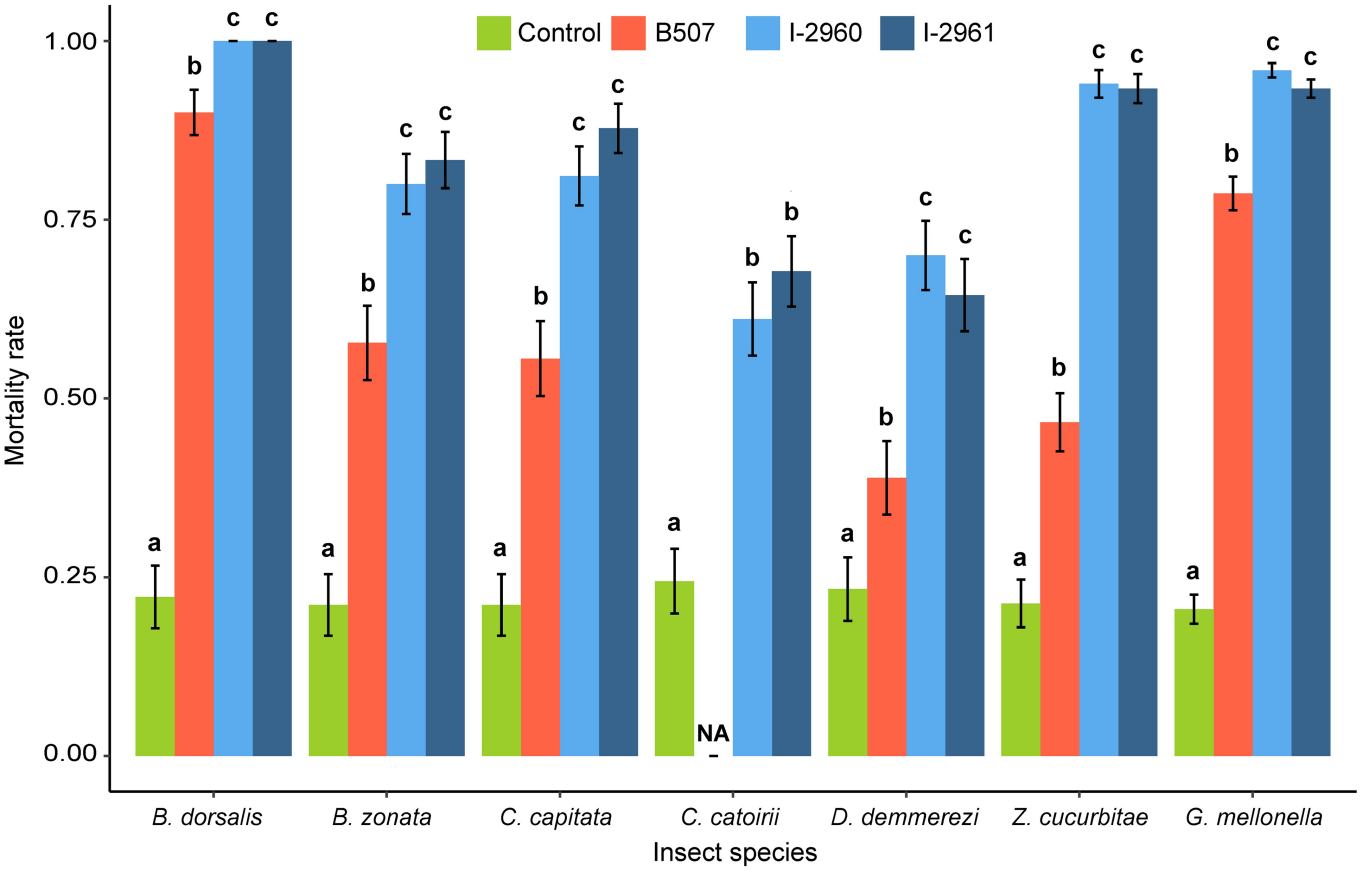
Mortality rate of six fruit fly species and *Galleria mellonella* treated with *Beauveria hoplocheli* strain B507, *B*. *bassiana* strains I-2960 and I-2961 using 10^6^ conidia.mL^-1^ suspensions. Data presented are means ± SEM, with three replicates of 30 insects for each treatment and each species. Mortality rates were calculated at the time when the mortality rate of the control reached 0.2. For each insect species, a generalized linear model was fitted and pairwise between treatment differences were tested using a likelihood ratio test. Different letters indicate significant differences between treatments (P < 0.05).

The analysis of mortality rates revealed that the effects of treatment (F = 36.76; DF = 3, 81; P < 0.0001), insect species (F = 8.62; DF = 5, 81; P < 0.0001), as well as the interaction insect species x treatment (F = 3.55; DF = 17, 81; P < 0.0001) were highly significant for the six fruit fly species and *G*. *mellonella* treated with 10^6^ conidia.mL^-1^. The analysis of mortality rates also revealed that the effects of the treatment (F = 53.28; DF = 3, 25; P < 0.0001), the species (F = 45.69; DF = 2, 25; P < 0.0001) and the insect species x treatment interaction (F = 29.00; DF = 6, 25; P < 0.0001) were highly significant for the species *H*. *marginalis*, *A*. *diaperinus* and *G*. *mellonella* treated with 10^8^ conidia.mL^-1^. The highly significant interaction between insect species and treatment shows that the differences between treatments depend on the insect species. Consequently, to compare the treatments, the insect mortality rate and survival were analysed independently for each species. The three *Beauveria* strains used at 10^6^ conidia.mL^-1^ were pathogenic to all the fruit fly species tested and to *G*. *mellonella* larvae as shown by the mortality rate and Kaplan-Meier survival curves, which differed significantly from the controls, irrespective of the *Beauveria* strain used (Figs 1 and 2). The mortality rate and the Kaplan-Meier survival analysis revealed differences in virulence between the *Beauveria* strains. *B*. *bassiana* strains I-2960 and I-2961 were significantly more virulent than the *B*. *hoplocheli* strain B507 for all fruit flies tested using the Kaplan-Meier survival analysis (except for *B*. *zonata*) and mortality rates (Figs 1 and 2). This result is also illustrated by the mean survival times estimated from the Kaplan-Meier survival analysis (S1 Table). Strains I-2960 and I-2961 exhibited a very similar virulence pattern for the different fruit flies (Figs 1 and 2 and S1 Table). The mortality rate and the Kaplan-Meier survival analysis of *G*. *mellonella* treated with 10^6^ conidia.mL^-1^ showed that B507 was significantly less virulent than *B*. *bassiana* strains (Figs 1 and 2). The Kaplan-Meier survival analysis showed that I-2961 was significantly less virulent than I-2960 at 10^6^ conidia.mL^-1^ (Fig 1).

**Fig 3:**
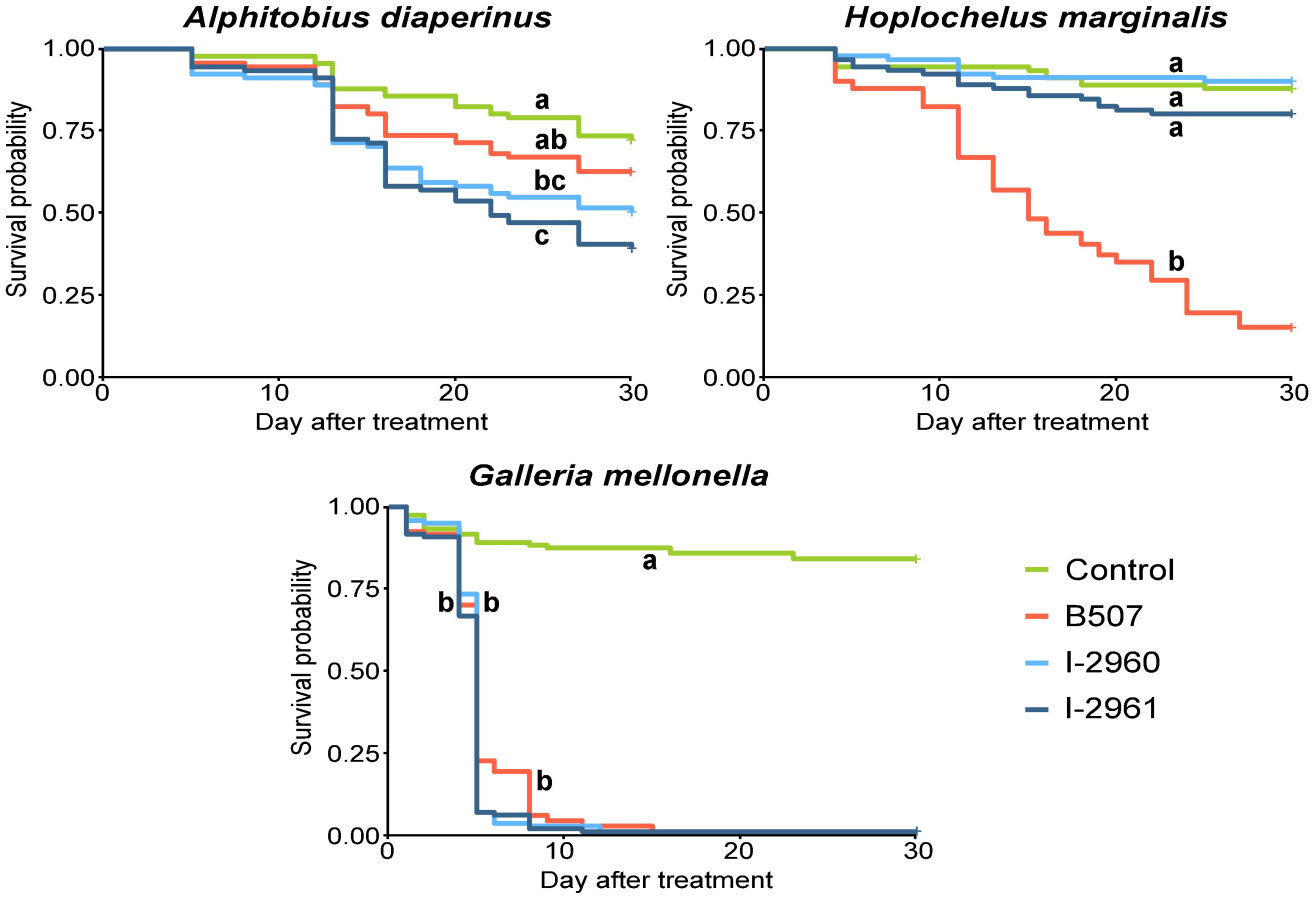
Kaplan-Meier survival curves for *Alphitobius diaperinus*, *Hoplochelus marginalis* and *Galleria mellonella* treated with *Beauveria hoplocheli* strain B507 and *B*. *bassiana* strains I-2960 and I-2961 using 10^8^ conidia.mL^-1^ suspensions. Different letters indicate significant differences between treatments within an insect species (log-rank test, P < 0.05 after Sidak’s correction). Crosses indicate censored data.

**Fig 4:**
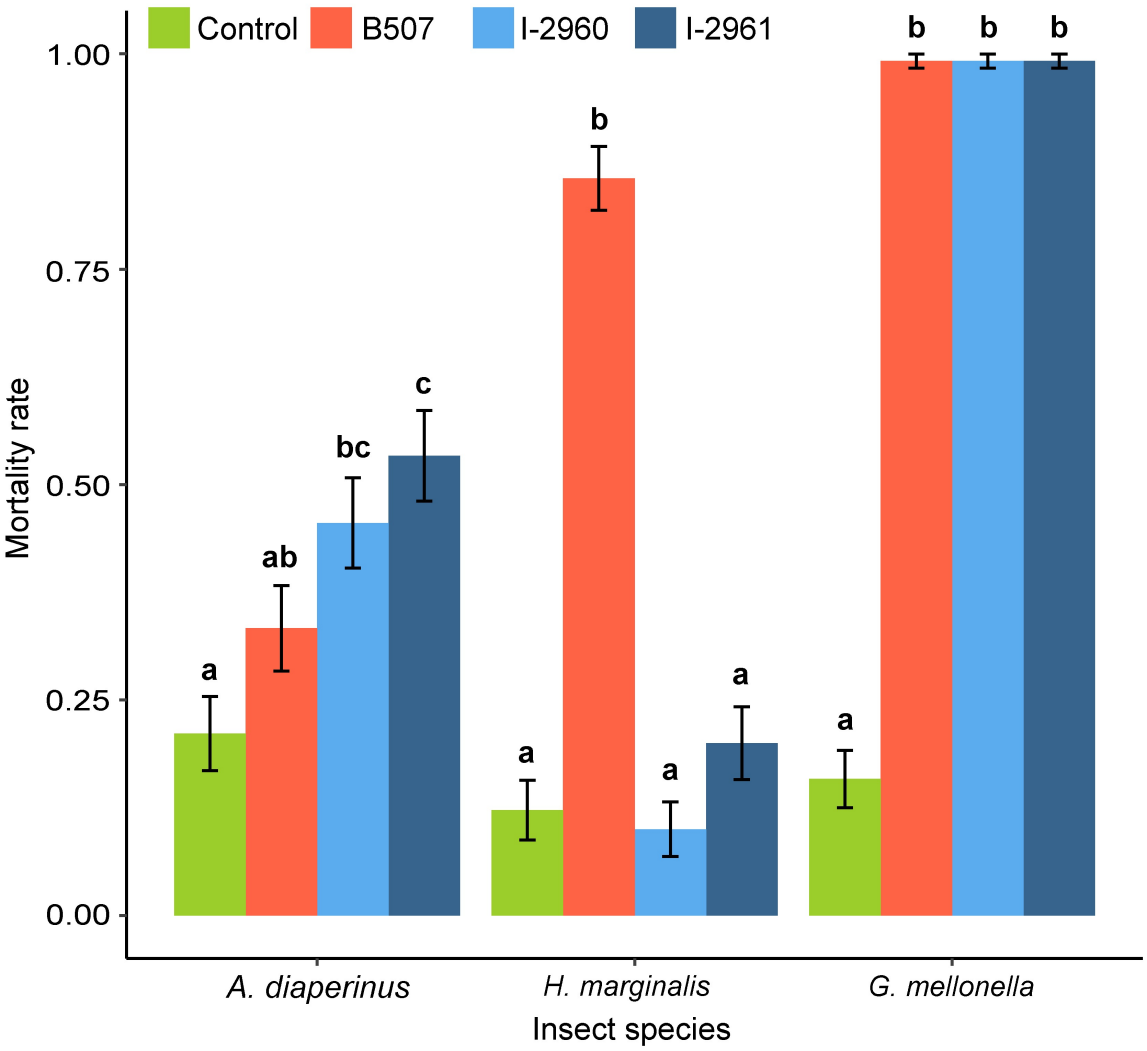
Mortality rate of *Alphitobius diaperinus*, *Hoplochelus marginalis* and *Galleria mellonella* treated with *Beauveria hoplocheli* strain B507, *B*. *bassiana* strains I-2960 and I-2961 using 10^8^ conidia.mL^-1^ suspensions. Data presented are means ± SEM, with three replicates of 30 insects for each treatment and each species. Mortality rates were calculated at the time when the mortality rate of the control reached 0.2. For each insect species, a generalized linear model was fitted and pairwise between treatment differences were tested using a likelihood ratio test. Different letters indicate significant differences between treatments (P < 0.05).

The three strains demonstrated a similar high level of virulence on *G*. *mellonella* at 10^8^ conidia.mL^-1^ (Figs 3 and 4 and S1 Table). *B*. *hoplocheli* strain B507 was the only strain pathogenic to the white grub *H*. *marginalis* at the tested dose of 10^8^ conidia.mL^-1^. The mortality rate and the Kaplan-Meier survival curves of *H*. *marginalis* larvae treated with the two *B*. *bassiana* strains were not significantly different from the control (Figs 3 and 4). The two *B*. *bassiana* strains were pathogenic to *A*. *diaperinus* larvae, resulting in mortality rates and Kaplan-Meier survival curves that were significantly different from the control (Figs 3 and 4). When *A*. *diaperinus* larvae were treated with strain B507, the mortality rate and the Kaplan-Meier survival curve were not significantly different from the control (Figs 3 and 4).

### Analysis of mycosis rate

**Fig 5:**
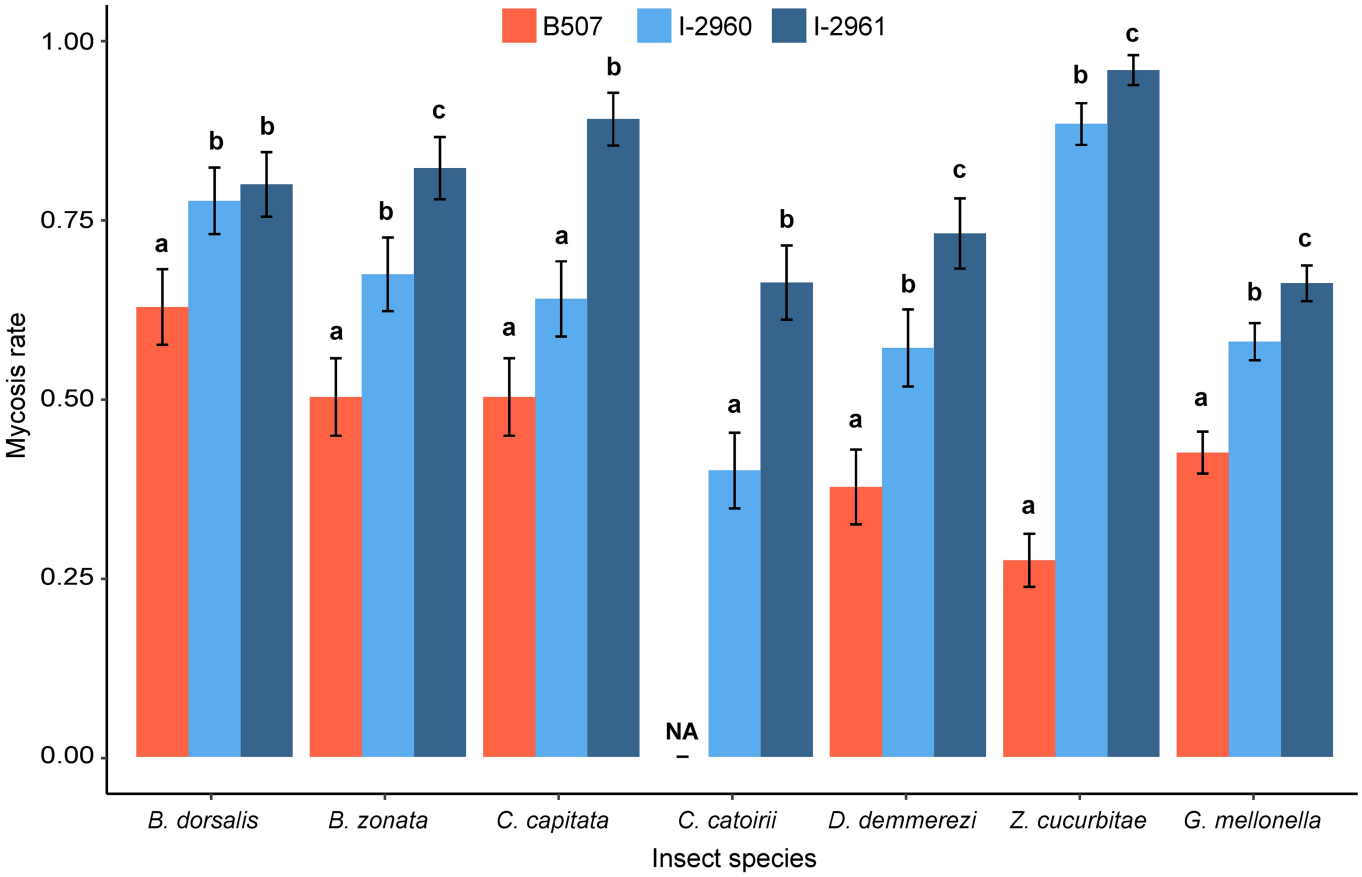
Mycosis rate of six fruit fly species and *Galleria mellonella* treated with *Beauveria hoplocheli* strain B507 and *B*. *bassiana* strains I-2760 and I-2761 using 10^6^ conidia.mL^-1^ suspensions. Data presented are means ± SEM, with three replicates of 30 insects for each treatment and each species. Mycosis rates were calculated using the percentage of cadavers showing external fungal growth out of the total number of tested insects. For each insect species, a generalized linear model was fitted and pairwise between treatment differences were tested using a likelihood ratio test. Different letters indicate significant differences between treatments (P < 0.05).

The analysis of the mycosis rate revealed that the treatments (F = 31.60; DF = 2, 55; P < 0.0001), and the insect species x treatment interaction (F = 2.08; DF = 11, 55; P = 0.038) had a significant effect for the six fruit fly species and *G*. *mellonella* treated with 10^6^ conidia.mL^-1^, while the insect species’ effect was not significant (F = 0.66; DF = 5, 55; P = 0.66). No mycosis was recorded on the control insects. The mycosis rate induced by the *B*. *hoplocheli* strain B507 was significantly lower than the rates induced by the two *B*. *bassiana* strains for four out of five of the fruit flies tested and for *G*. *mellonella* (Fig 5). The *B*. *bassiana* strain I-2961 induced significantly higher mycosis rates than strain I-2960 for all fruit flies tested (except *B*. *dorsalis*) and *G*. *mellonella*, although the mortality rates caused by both strains were similar (Figs 1, 2 and 5). In the case of *H*. *marginalis*, the *B*. *hoplocheli* strain caused a mycosis rate of 0.27 ± 0.05 for a mortality rate of 0.86 ± 0.04. None of the white grubs that died after treatment with strain I-2960 developed external fungal growth. For strain I-2961, the mycosis rate was 0.04 ± 0.02, which was significantly different from the control (Chi^2^ = 5.66; DF = 1; P = 0.0174). No mycosis was recorded on *A*. *diaperinus* larvae, irrespective of the *Beauveria* strain considered.

## Discussion

We demonstrated that there are significant differences in the physiological host range of the three *Beauveria* strains tested. The *B*. *bassiana* strains and the *B*. *hoplocheli* strain express different pathogenicity patterns across several insects belonging to the Coleoptera order. *B*. *bassiana* strains killed *A*. *diaperinus*, but were not pathogenic to *H*. *marginalis*, although they are both beetle larval stages. These findings confirmed the results of previous studies, which showed that the strain I-2960 was not pathogenic to *H*. *marginalis* [24, 42]. Few studies have reported an absence of pathogenicity for *B*. *bassiana* strains. It is interesting to note that the work by Maurer *et al*. [43] shows that several *B*. *bassiana* strains that were isolated from insects other than *Ostrinia nubilalis*, were not pathogenic to this species. Strain I-2961 did not cause a significant increase in *H*. *marginalis* mortality rate, although it produced mycosis on a few individuals. It is possible that the fungus was able to complete its biological cycle after overcoming the insect’s defences. The *B*. *hoplocheli* strain B507 was pathogenic to *H*. *marginalis*, but was not pathogenic to *A*. *diaperinus*. Neuvéglise *et al*. [24] also found that several *B*. *hoplocheli* strains were not pathogenic to *M*. *melolontha*, a beetle belonging to the Melolonthinae subfamily, the same subfamily as *H*. *marginalis*. These results confirm that, to date, *B*. *hoplocheli* is the only available BCA for controlling *H*. *marginalis* in sugarcane fields.

The *B*. *hoplocheli* strain, B507, was pathogenic to the tested fruit flies and to the greater wax moth, but was less virulent than the two *B*. *bassiana* strains. Such differences in virulence have been observed at the inter- and the intra-species level for *Beauveria*. At the inter-species level, Goble *et al*. [44] showed that *B*. *brongniartii* isolates were less effective against the Asian long-horned beetle than other Hypocreales fungal species, including *Beauveria asiatica*. Differences in virulence have also been reported at the intra-species level [33, 34, 45]. Differences in virulence observed among the *Beauveria* strains could be linked to conidial attachment on the cuticle, germination, as well as strategies to evade the host’s immune system [46]. In addition, virulence is affected by factors including cuticle-degrading enzymes or toxic proteins, which are produced by the fungus [31, 47]. The main difference between the two *B*. *bassiana* strains was their potential to cause mycosis. Strain I-2961 sporulated significantly more on fruit fly cadavers than I-2960. The two *B*. *bassiana* strains were pathogenic to *A*. *diaperinus* larvae, but no mycosis was observed. Fungal development on host cadavers is a crucial parameter for BCA selection because the effectiveness of insect population control depends on the fungus’ capacity to complete its biological cycle and transmission to other insects [48]. Many factors might affect the sporulation on cadavers, including temperature, humidity, conidia number and insect age [49, 50].

Using laboratory bioassays to characterize their physiological host range, we demonstrated that *B*. *hoplocheli* strain B507 and the two *B*. *bassiana* strains I-2961 and I-2960 can infect a wide range of insects belonging to three different orders. Strain I-2961 and, to a lesser extent, strain I-2960, were also known pathogens of the Coleoptera *Rhynchophorus ferrugineus* [51, 52]. Strain I-2960 showed pathogenicity toward the lepidopteran pests *Ostrinia nubilalis*, *Paysandisia archon* and *Thaumetopoea pityocampa* [53, 54]. This broad host range means that both *B*. *bassiana* strains have great potential to control diverse pests. As yet, the *B*. *hoplocheli* strain B507 has only been used to control the white grub *H*. *marginalis*, but we have shown that this species is not specific to Coleoptera and can infect Diptera and Lepidoptera. Hu *et al*. [55] suggested that speciation in the *Metarhizium* genus was closely related to host specificity, with an evolutionary route going from specialists to generalists, via intermediate host range species. Further pathogenicity and genomic studies are required to determine whether such a speciation pattern exists in *Beauveria*. However, as in the *Metarhizium* genus, it seems that most *Beauveria* species have a broad host range, which is probably linked to ecological fitness [55].

When these entomopathogenic fungal strains are used as BCA, their broad host range could be a concern in terms of their impact on non-target species. The ecological host range should be considered, as it is not unusual that hosts infected in the laboratory have never been found infected in the field [4]. Hypocreales fungi, such as *Beauveria* spp., are facultative insect pathogens capable of saprophytic and endophytic life stages. Therefore, soil characteristics, abiotic factors, plant species, as well as agricultural practices can have a great impact on their persistence and activity [56–58]. There is some evidence that *B*. *bassiana* strains may be adapted to a habitat type rather than to a particular host [59]. When choosing a suitable BCA, it is important to study the physiological host range, combined with an assessment of the impact that environmental conditions have on the fungal strain’s development. An evaluation of the persistence and distribution of the introduced biocontrol agent *B*. *hoplocheli* throughout Réunion is currently underway. This research will help shed light on the factors influencing its effectiveness and impact.

## Supporting information

S1 Table Mean survival time in days of insects treated with *Beauveria hoplocheli* strain B507 and *Beauveria bassiana* strains I-2960 and I-2961 at 10^6^ conidia.mL^-1^ or 10^8^ conidia.mL^-1^. Kaplan-Meier analysis was used to estimate the mean survival time for each *Beauveria* strain in each bioassay (three replicates of 30 insects).
(DOCX)

## References

[1] Brodeur J. Host specificity in biological control: insights from opportunistic pathogens. Evol Appl. 2012;5(5):470–80. doi: 10.1111/j.1752-4571.2012.00273.x 22949922 10.1111/j.1752-4571.2012.00273.x PMC3407865

[2] Van Lenteren JC, Bale J, Bigler F, Hokkanen HMT, Loomans AJM. Assessing risks of releasing exotic biological control agents of arthropod pests. Annu Rev Entomol. 2006;51:609–34. doi: 10.1146/annurev.ento.51.110104.151129 16332225 10.1146/annurev.ento.51.110104.151129

[3] Glare T, Caradus J, Gelernter W, Jackson T, Keyhani N, Köhl J, et al Have biopesticides come of age? Trends Biotechnol. 2012;30(5):250–8. doi: 10.1016/j.tibtech.2012.01.003 22336383 10.1016/j.tibtech.2012.01.003

[4] Hajek AE, Goettel MS. Guidelines for evaluating effects of entomopathogens on non-target organisms In: Lacey LA, Kaya HK, editors. Field Manual of Techniques in Invertebrate Pathology. Dordrecht: Springer Netherlands; 2007 p. 816–33.

[5] Fargues J, Remaudiere G. Considerations on the specificity of entomopathogenic fungi. Mycopathologia. 1977;62(1):31–7.

[6] Goettel M, Poprawski T, Vandenberg J, Li Z, Roberts D. Safety to nontarget invertebrates of fungal biocontrol agents In: Laird M, Lacey L, Davidson E, editors. Safety of microbial insecticides: CRC Press; 1990 p. 209–29.

[7] Van Driesche R, Hoddle M. Non-target effects of insect biocontrol agents and trends in host specificity since 1985. CAB Reviews. 2016;11(44):1–66.

[8] Shapiro-Ilan DI, Fuxa JR, Lacey LA, Onstad DW, Kaya HK. Definitions of pathogenicity and virulence in invertebrate pathology. J Invertebr Pathol. 2005;88(1):1–7. doi: 10.1016/j.jip.2004.10.003 15707863 10.1016/j.jip.2004.10.003

[9] Onstad D, Fuxa J, Humber R, Oestergaard J, Shapiro-Ilan D, Gouli V, et al An abridged glossary of terms used in invertebrate pathology 2006 http://www.sipweb.org/resources/glossary.html.

[10] Inglis GD, Enkerli J, Goettel MS. Laboratory techniques used for entomopathogenic fungi: Hypocreales In: Lacey LA, editor. Manual of techniques in invertebrate pathology. 2nd ed London, UK: Academic Press; 2012 p. 189–253.

[11] Faria MRd Wraight SP. Mycoinsecticides and mycoacaricides: a comprehensive list with worldwide coverage and international classification of formulation types. Biol Control. 2007;43(3):237–56.

[12] Pell JK, Eilenberg J, Hajek AE, Steinkraus DC. Biology, ecology and pest management potential of Entomophthorales In: Butt TM, Jackson C, Magan N, editors. Fungi as biocontrol agents: progress, problems and potential. Wallingford, UK: CAB International; 2001 p. 71–153.

[13] Jensen AB, Thomsen L, Eilenberg J. Value of host range, morphological, and genetic characteristics within the *Entomophthora muscae* species complex. Mycol Res. 2006;110(8):941–50. 16905302 10.1016/j.mycres.2006.06.003

[14] Gryganskyi A, Humber R, Smith M, Hodge K, Huang B, Voigt K, et al Phylogenetic lineages in Entomophthoromycota. Persoonia. 2013;30:94–105. doi: 10.3767/003158513X666330 24027349 10.3767/003158513X666330 PMC3734969

[15] Hajek AE, Gardescu S, Delalibera Jr. I. Classical biological control of insects and mites: a worldwide catalogue of pathogen and nematode introductions. Morgantown, WV: FHTET, USDA Forest Service; 2016. 57 p.

[16] Feng M, Poprawski T, Khachatourians GG. Production, formulation and application of the entomopathogenic fungus *Beauveria bassiana* for insect control: current status. Biocontrol Sci Technol. 1994;4(1):3–34.

[17] Shah PA, Pell JK. Entomopathogenic fungi as biological control agents. Appl Microbiol Biotechnol. 2003;61(5–6):413–23. doi: 10.1007/s00253-003-1240-8 12764556 10.1007/s00253-003-1240-8

[18] Vestergaard S, Cherry A, Keller S, Goettel M. Safety of hyphomycete fungi as microbial control agents In: Hokkanen HMT, Hajek AE, editors. Environmental impacts of microbial insecticides: need and methods for risk assessment. Progress in Biological Control. Dordrecht, Netherlands: Springer; 2003 p. 35–62.

[19] Imoulan A, Hussain M, Kirk PM, El Meziane A, Yao Y-J. Entomopathogenic fungus *Beauveria*: Host specificity, ecology and significance of morpho-molecular characterization in accurate taxonomic classification. J Asia Pac Entomol. 2017;20:1204–12.

[20] Leatherdale D. The arthropod hosts of entomogenous fungi in Britain. BioControl. 1970;15(4):419–35.

[21] Rehner SA, Minnis AM, Sung GH, Luangsa-ard JJ, Devotto L, Humber RA. Phylogeny and systematics of the anamorphic, entomopathogenic genus *Beauveria*. Mycologia. 2011;103(5):1055–73. doi: 10.3852/10-302 21482632 10.3852/10-302

[22] Robène-Soustrade I, Jouen E, Pastou D, Payet-Hoareau M, Goble TA, Linderme D, et al Description and phylogenetic placement of *Beauveria hoplocheli* sp. nov. used in the biological control of the sugarcane white grub, *Hoplochelus marginalis*, in Reunion Island. Mycologia. 2015;107(6):1221–32. doi: 10.3852/14-344 26297783 10.3852/14-344

[23] Vercambre B, Goebel O, Riba G, Marchal M, Neuvéglise C, Ferron P. Success in biological control of a soil pest, *Hoplochelus marginalis*, in Reunion island: choice of a suitable fungus VIth International Colloquium on Invertebrate Pathology and Microbial Control; 1993 12 7–9; Montpellier, France: Society for Invertebrate Pathology; 1994. p. 283–8.

[24] Neuvéglise C, Brygoo Y, Riba G. 28s rDNA group-I introns: a powerful tool for identifying strains of *Beauveria brongniartii*. Mol Ecol. 1997;6(4):373–81. 9131812 10.1046/j.1365-294x.1997.00202.x

[25] Bricca ES, Nibouche S, Delatte H, Normand F, Amouroux P. Test of the pathogenicity of two commercial *Beauveria* strains on third-instar larvae of the mango blossom gall midge, *Procontarinia mangiferae* (Felt)(Diptera: Cecidomyiidae). Fruits. 2014;69(3):189–94.

[26] Quesada-Moraga E, Vey A. Intra-specific variation in virulence and in vitro production of macromolecular toxins active against locust among *Beauveria bassiana* strains and effects of in vivo and in vitro passage on these factors. Biocontrol Sci Technol. 2003;13(3):323–40.

[27] Quesada-Moraga E, Landa B, Muñoz-Ledesma J, Jiménez-Diáz R, Santiago-Alvarez C. Endophytic colonisation of opium poppy, *Papaver somniferum*, by an entomopathogenic *Beauveria bassiana* strain. Mycopathologia. 2006;161(5):323–9. doi: 10.1007/s11046-006-0014-0 16649082 10.1007/s11046-006-0014-0

[28] Valero-Jiménez CA, Debets AJ, van Kan JA, Schoustra SE, Takken W, Zwaan BJ, et al Natural variation in virulence of the entomopathogenic fungus *Beauveria bassiana* against malaria mosquitoes. Malar J. 2014;13(1):1–8. 25480526 10.1186/1475-2875-13-479 PMC4364330

[29] Carneiro AA, Gomes EA, Guimarães CT, Fernandes FT, Carneiro NP, Cruz I. Molecular characterization and pathogenicity of isolates of *Beauveria* spp. to fall armyworm. Pesq Agropec Bras. 2008;43(4):513–20.

[30] Talaei-Hassanloui R, Kharazi-Pakdel A, Goettel M, Mozaffari J. Variation in virulence of *Beauveria bassiana isolates* and its relatedness to some morphological characteristics. Biocontrol Sci Technol. 2006;16(5):525–34.

[31] Zare M, Talaei-Hassanloui R, Fotouhifar K-B. Relatedness of proteolytic potency and virulence in entomopathogenic fungus *Beauveria bassiana* isolates. J Crop Prot. 2014;3(4):425–34.

[32] Todorova S, Côté J-C, Martel P, Coderre D. Heterogeneity of two *Beauveria bassiana* strains revealed by biochemical tests, protein profiles and bio-assays of *Leptinotarsa decemlineata* (Col.: Chrysomelidae) and *Coleomegilla macultata lengi* (Col.: Coccinellidae) larvae. Entomophaga. 1994;39(2):159–69.

[33] Wraight SP, Ramos ME, Avery PB, Jaronski ST, Vandenberg JD. Comparative virulence of *Beauveria bassiana* isolates against lepidopteran pests of vegetable crops. J Invertebr Pathol. 2010;103(3):186–99. doi: 10.1016/j.jip.2010.01.001 20060396 10.1016/j.jip.2010.01.001

[34] Uma Devi K, Padmavathi J, Uma Maheswara Rao C, Khan AAP, Mohan MC. A study of host specificity in the entomopathogenic fungus *Beauveria bassiana* (Hypocreales, Clavicipitaceae). Biocontrol Sci Technol. 2008;18(10):975–89.

[35] Mze Hassani I, Raveloson-Ravaomanarivo LH, Delatte H, Chiroleu F, Allibert A, Nouhou S, et al Invasion by *Bactrocera dorsalis* and niche partitioning among tephritid species in Comoros. Bull Entomol Res. 2016;106(6):749–58. doi: 10.1017/S0007485316000456 27312045 10.1017/S0007485316000456

[36] Duyck PF, Quilici S. Survival and development of different life stages of three *Ceratitis* spp. (Diptera: Tephritidae) reared at five constant temperatures. Bull Entomol Res. 2002;92(6):461–9. 17598297 10.1079/ber2002188

[37] Meyling NV. Methods for isolation of entomopathogenic fungi from the soil environment. Laboratory manual. University of Copenhagen; 2007.

[38] Ekesi S, Maniania N, Lux S. Mortality in three African tephritid fruit fly puparia and adults caused by the entomopathogenic fungi, *Metarhizium anisopliae* and *Beauveria bassiana*. Biocontrol Sci Technol. 2002;12(1):7–17.

[39] Quesada-Moraga E, Ruiz-García A, Santiago-Álvarez C. Laboratory Evaluation of Entomopathogenic Fungi *Beauveria bassiana* and *Metarhizium anisopliae* Against Puparia and Adults of *Ceratitis capitata* (Diptera: Tephritidae). J Econ Entomol. 2006;99(6):1955–66. 17195660 10.1603/0022-0493-99.6.1955

[40] De la Rosa W, Lopez F, Liedo P. *Beauveria bassiana* as a pathogen of the Mexican fruit fly (Diptera: Tephritidae) under laboratory conditions. J Econ Entomol. 2002;95(1):36–43. 11942762 10.1603/0022-0493-95.1.36

[41] SAS Institute. SAS/STAT. 9.3 ed. Cary, NC, USA: SAS Institute Inc.; 2010.

[42] Neuvéglise C, Brygoo Y, Vercambre B, Riba G. Comparative analysis of molecular and biological characteristics of strains of *Beauveria brongniartii* isolated from insects. Mycol Res. 1994;98(3):322–8.

[43] Maurer P, Couteaudier Y, Girard P, Bridge P, Riba G. Genetic diversity of *Beauveria bassiana* and relatedness to host insect range. Mycol Res. 1997;101(2):159–64.

[44] Goble TA, Rehner SA, Long SJ, Gardescu S, Hajek AE. Comparing virulence of North American *Beauveria brongniartii* and commercial pathogenic fungi against Asian longhorned beetles. Biol Control. 2014;72:91–7.

[45] Keller S, Schweizer C, Shah P. Differential susceptibility of two Melolontha populations to infections by the fungus *Beauveria brongniartii*. Biocontrol Sci Technol. 1999;9(3):441–6.

[46] Lu H-L, St. Leger R. Insect immunity to entomopathogenic fungi. Adv Genet. 2016;94:251–85. doi: 10.1016/bs.adgen.2015.11.002 27131327 10.1016/bs.adgen.2015.11.002

[47] Ortiz-Urquiza A, Riveiro-Miranda L, Santiago-Alvarez C, Quesada-Moraga E. Insect-toxic secreted proteins and virulence of the entomopathogenic fungus *Beauveria bassiana*. J Invertebr Pathol. 2010;105(3):270–8. doi: 10.1016/j.jip.2010.07.003 20674578 10.1016/j.jip.2010.07.003

[48] Hajek AE, St. Leger RJ. Interactions between fungal pathogens and insect hosts. Annu Rev Entomol. 1994;39(1):293–322.

[49] Fargues J, Luz C. Effects of fluctuating moisture and temperature regimes on sporulation of *Beauveria bassiana* on cadavers of *Rhodnius prolixus*. Biocontrol Sci Technol. 1998;8(3):323–34.

[50] Tefera T, Pringle K. Effect of exposure method to *Beauveria bassiana* and conidia concentration on mortality, mycosis, and sporulation in cadavers of *Chilo partellus* (Lepidoptera: Pyralidae). J Invertebr Pathol. 2003;84(2):90–5. 14615217 10.1016/j.jip.2003.08.001

[51] European Food Safety Authority. Peer review of the pesticide risk assessment of the active substance *Beauveria bassiana* strain NPP111B005. EFSA Journal. 2015;13(10):4264.

[52] European Food Safety Authority. Peer review of the pesticide risk assessment of the active substance *Beauveria bassiana* strain 147. EFSA Journal. 2015;13(10):4261.

[53] Besse S, Bonhomme A, Panchaud K. Preventive and curative efficacy of Ostrinil^®^, a Beauveria bassiana spores based microgranule formulation, against the Castniid palm borer Paysandisia archon (Burmeister, 1880). 3rd Annual Biocontrol Industry Meeting; 2008 Oct 20–21; Lucerne, Switzerland.

[54] Bonnet C, Martin J, Mazet R, Correard M, Besse S. Beauveria bassiana (Bals.-Criv) Vuillemin: an entomopathogen to reduce the expansion of the pine processionary plants transported by container. 3e Conférence sur l’entretien des Zones Non Agricoles; 2013 Oct 15–17; Toulouse, France: Association Française de Protection des Plantes (AFPP); 2013. p. 209–13. French.

[55] Hu X, Xiao G, Zheng P, Shang Y, Su Y, Zhang X, et al Trajectory and genomic determinants of fungal-pathogen speciation and host adaptation. Proc Natl Acad Sci U S A. 2014;111(47):16796–801. doi: 10.1073/pnas.1412662111 25368161 10.1073/pnas.1412662111 PMC4250126

[56] Quesada-Moraga E, Navas-Cortés JA, Maranhao EA, Ortiz-Urquiza A, Santiago-Álvarez C. Factors affecting the occurrence and distribution of entomopathogenic fungi in natural and cultivated soils. Mycol Res. 2007;111(8):947–66. 17766099 10.1016/j.mycres.2007.06.006

[57] Meyling NV, Lubeck M, Buckley EP, Eilenberg J, Rehner SA. Community composition, host range and genetic structure of the fungal entomopathogen *Beauveria* in adjoining agricultural and seminatural habitats. Mol Ecol. 2009;18(6):1282–93. doi: 10.1111/j.1365-294X.2009.04095.x 19226319 10.1111/j.1365-294X.2009.04095.x

[58] Moonjely S, Barelli L, Bidochka MJ. Insect pathogenic fungi as endophytes. Adv Genet. 2016;94:107–35. doi: 10.1016/bs.adgen.2015.12.004 27131324 10.1016/bs.adgen.2015.12.004

[59] Bidochka MJ, Menzies FV, Kamp AM. Genetic groups of the insect-pathogenic fungus *Beauveria bassiana* are associated with habitat and thermal growth preferences. Arch Microbiol. 2002;178(6):531–7. doi: 10.1007/s00203-002-0490-7 12420176 10.1007/s00203-002-0490-7

