# Supplementary material for: Variation in physiological host range in three strains of two species of the entomopathogenic fungus Beauveria
Source: PLoS One. 2018 Jul 5;13(7):e0199199. doi: 10.1371/journal.pone.0199199 (PMC6033404; doi:10.1371/journal.pone.0199199)
Supplement: S1 Table — Kaplan-Meier analysis was used to estimate the mean survival time for each Beauveria strain in each bioassay (three replicates of 30 insects). (DOCX) [file pone.0199199.s001.docx]

**S1 Table**. **Mean survival time in days of insects treated with *Beauveria hoplocheli* strain B507 and *Beauveria bassiana* strains I-2960 and I-2961, using 10^6^ conidia.mL^-1^ or 10^8^ conidia.mL^-1^ suspensions.**

| **Insect** | **Mean survival time (means ± standard error)** | | | |
| --- | --- | --- | --- | --- |
|  | **Control** | **B507** | **I-2960** | **I-2961** |
| **10^6^ conidia.mL^-1^** |  |  |  |  |
| *Bactrocera dorsalis* | 21.0 ± 1.0 | 9.1 ± 0.5 | 4.7 ± 0.2 | 5.2 ± 0.1 |
| *Bactrocera zonata* | 17.9 ± 1.1 | 8.1 ± 0.6 | 5.3 ± 0.2 | 5.2 ± 0.2 |
| *Ceratitis capitata* | 18.2 ± 0.8 | 11.4 ± 0.7 | 8.5 ± 0.5 | 7.6 ± 0.4 |
| *Ceratitis catoirii* | 16.6 ± 0.9 | NA | 10.1 ± 0.8 | 9.8 ± 0.7 |
| *Dacus demmerezi* | 19.0 ± 1.1 | 12.6  ± 0.9 | 9.6 ± 1.0 | 7.3 ± 0.5 |
| *Zeugodacus cucurbitae* | 26.5 ± 0.6 | 22.2 ± 0.7 | 9.9 ± 0.4 | 8.9 ± 0.4 |
| *Galleria mellonella* | 25.4 ± 0.5 | 12.8 ± 0.6 | 6.5 ± 0.3 | 8.7 ± 0.3 |
| **10^8^ conidia.mL^-1^** |  |  |  |  |
| *Alphitobius diaperinus* | 26.7 ± 0.7 | 24.5 ± 0.9 | 22.3 ± 0.9 | 21.5 ± 0.9 |
| *Hoplochelus marginalis* | 27.8 ± 0.7 | 16.9 ± 0.9 | 28.1 ± 0.7 | 26.4 ± 0.8 |
| *Galleria mellonella* | 26.3 ± 0.8 | 5.4 ± 0.3 | 5.0 ± 0.2 | 4.8 ± 0.3 |

Kaplan-Meier analysis was used to estimate the mean survival time for each *Beauveria* strains in each bioassay (three replicates of 30 insects).
